# Deaths during Sexual Activity

**DOI:** 10.15388/Amed.2024.31.1.8

**Published:** 2024-02-27

**Authors:** Agnė Okulevičiūtė, Sigitas Chmieliauskas, Sigitas Laima, Diana Vasiljevaitė, Jurgita Stasiūnienė

**Affiliations:** 1 Faculty of Medicine, Vilnius University, Vilnius, Lithuania.; 2 Department of Pathology, Forensic Medicine and Pharmacology, Institute of Biomedical Sciences of the Faculty of Medicine of Vilnius University, Vilnius, Lithuania

**Keywords:** deaths during sexual intercourse, autoerotic deaths, deaths during sexual activity, autopsy, forensic pathology, mirtys lytinių santykių metu, autoerotinės mirtys, mirtys seksualinės veiklos metu, autopsija, teismo medicina

## Abstract

**Background:**

Deaths during sexual activities are rarely identified phenomena in forensic medicine practice. Most often, such deaths are classified as accidents or deaths due to the manifestation of certain diseases during sexual activity. It is important to rule out homicide or suicide as the cause of death when investigating sexual deaths. Determining the cause of death requires a comprehensive assessment of the evidence and circumstances and should not be based solely on autopsy findings. When determining the cause of death, it is necessary to evaluate the circumstances of the discovery, important evidence found near the body, the position of the deceased, the place where the deceased was found, and the characteristics of the environment.

**Cases:**

*Case 1:* A 65-year-old male was clothed in women’s underwear and was found hanging in a noose in a bedroom after a house fire. The autopsy revealed a ligature mark on the neck, bruises in neck muscles, tears in carotid arteries, and signs of acute pulmonary distension. The cause of death was determined to be suffocation due to neck compression by a ligature, compounded by significant alcohol intoxication, with additional postmortem burns covering 30% of the body surface area likely occurring after death.

*Case 2:* A 55-year-old naked male was found without external injuries but with a plastic tube inserted into the rectum, causing a 2.5 cm rupture in the ileum. The perforation led to complications, including purulent diffuse peritonitis, intoxication, and acute cardiac and respiratory failure, resulting in death within 3–6 hours after insertion. Concurrent findings included atherosclerotic changes in the heart, internal organ hyperemia and edema, hepatic steatosis, renal cyst, and a lack of ethyl alcohol in blood but 0.17 ‰ presence in urine according to toxicology analysis.

**Conclusions:**

A detailed evaluation of all the evidence is very important in the forensic examination of the deceased during sexual activity. Therefore, to determine the cause of death, not only the autopsy data, toxicological and microscopic examinations of the deceased are important, but also the evaluation of all findings at the scene. The most common cause of death of an autoerotic nature is asphyxia, and the most commonly identified group of the dead are men aged around 40 years.

## Abstract (it)

**Įvadas:**

Mirtys seksualinio pobūdžio veiklų metu yra retai nustatomas reiškinys teismo medicinos praktikoje. Dažniausiai tokios mirtys yra skirstomos į nelaimingus atsitikimus ar mirtis sąlygotas tam tikrų ligų pasireiškimo seksualinės veiklos metu. Atliekant seksualinės veiklos metu mirusiųjų asmenų tyrimą, yra svarbu atmesti nužudymą ar savižudybę kaip mirties priežastį. Mirties priežasčiai nustatyti yra svarbus visapusiškas įkalčių ir aplinkybių vertinimas, todėl ji neturėtų būti grindžiama vien tik autopsijos duomenimis. Nustatant mirties priežastį yra būtina vertinti radimo aplinkybes, svarbūs šalia kūno rasti įkalčiai, mirusiojo padėtis, vieta kurioje rastas mirusysis bei aplinkos ypatumai.

**Atvejai:**

*1 atvejis*: užgesinus gaisrą namuose, buvo rastas miegamajame, apsirengęs moteriškais drabužiais, 65 metų vyro lavonas, kabantis kilpoje. Autopsijos metu nustatyta stranguliacinė vaga kakle, kraujosruvos kaklo raumenyse, miego arterijų sienelių pažeidimai, veninė vidaus organų pilnakraujystė bei plaučių pabrinkimas. Nustatyta mirties priežastis uždusimas suspaudus kaklą su smaugvirve. Toksikologinio tyrimo metu vidaus terpėse nustatytas etilo alkoholis. Papildomai nustatyti pomirtiniai kūno nudegimai apimantys 30 % kūno paviršiaus ploto.

*2 atvejis*: 55 metų vyro lavonas rastas nuogas, namo tualete, be išorinių sužalojimų, tačiau su plastikiniu vamzdeliu tiesiojoje žarnoje. Autopsijos metu nustatytas klubinėje žarnoje 2,5 cm ilgio plyšimas, kuris komplikavosi difuziniu peritonitu, intoksikacija, ūminiu širdies bei kvėpavimo nepakankamumu. Remiantis tyrimo duomenimis mirtis įvyko per 3–6 valandas nuo žarnos sienelės plyšimo. Toksikologinio tyrimo duomenimis nustatyta šlapime 0,17 ‰ etilo alkoholio koncentracija.

**Išvada:**

Detalus visų įkalčių įvertinimas yra labai svarbus atliekant mirusiųjų seksualinės veiklos metu teismo medicinos ekspertizę. Todėl mirties priežasčiai nustatyti yra svarbūs ne tik mirusiojo autopsijos duomenys, toksikologiniai bei mikroskopiniai tyrimai, tačiau ir įvykio vietos visų radinių vertinimas. Dažniausia autoerotinio pobūdžio mirties priežastis yra asfiksija, bei dažniausiai nustatoma mirusiųjų grupė yra apie 40 metų amžiaus vyriškos lyties asmenys.

## Introduction

During forensic examination of deaths during sexual activity, it is important to rule out homicide or suicide. A comprehensive approach involving multiple diagnostic techniques is advisable, and relying solely on autopsy results should be avoided when making a diagnosis. When determining the cause of death, it is necessary to evaluate the circumstances of the discovery, important evidence found near the body, the position of the deceased, the place where the deceased was found, and the characteristics of the environment. The purpose of the study is to present two rarely identified cases in practice during sexual activity, which demonstrate the importance of examining the scene during each investigation, with the help of which the circumstances surrounding the death could be revealed and to review the literature on this topic. After the investigation of the considered cases, it was found that both cases are considered accidents during autoerotic activities.

## Methods

### Study design and data source

The performed retrospective study included 2 victims, whose cause of death was during sexual activity. The information concerning the examination of deceased individuals after their death was sourced from the database of the Lithuanian State Forensic Medicine Service. After the analysis of the deceased persons in the years 2015–2022 in the State Forensic Medicine Service, out of 1638 deceased persons, only two cases meeting the criteria of autoerotic deaths were identified.

A complete forensic pathology autopsy was performed on all deceased individuals. In each instance, details were provided by law enforcement agencies, encompassing the incident location, time of death, and the likely cause of death.

### Identification of cases

The study involved 2 cases, where the death was caused by autoerotic events. All the victims died suddenly and did not receive any medical treatment. Cases, where the cause of death could not be determined due to the lack of sexual activity evidence, were excluded from the analysis. All the cases were identified as instances of autoerotic fatalities.

### Toxicological methods

In all cases, the deceased were subjected to complete autopsies with tests for ethyl alcohol and its surrogates as well as toxicological tests for the detection of drugs and other potent substances in the blood and urine. Headspace gas chromatography was used for alcohol detection.

### Histological methods

Histological sections were cut and prepared for routine light microscopy. Histomorphological features of the samples were examined using haematoxylin and eosin (H&E) staining. Perls’ Prussian blue reaction was used to detect ferric iron, and Masson’s trichrome staining was used for collagen fibers.

## Practical examples

### Case 1

**Fig. 1:**
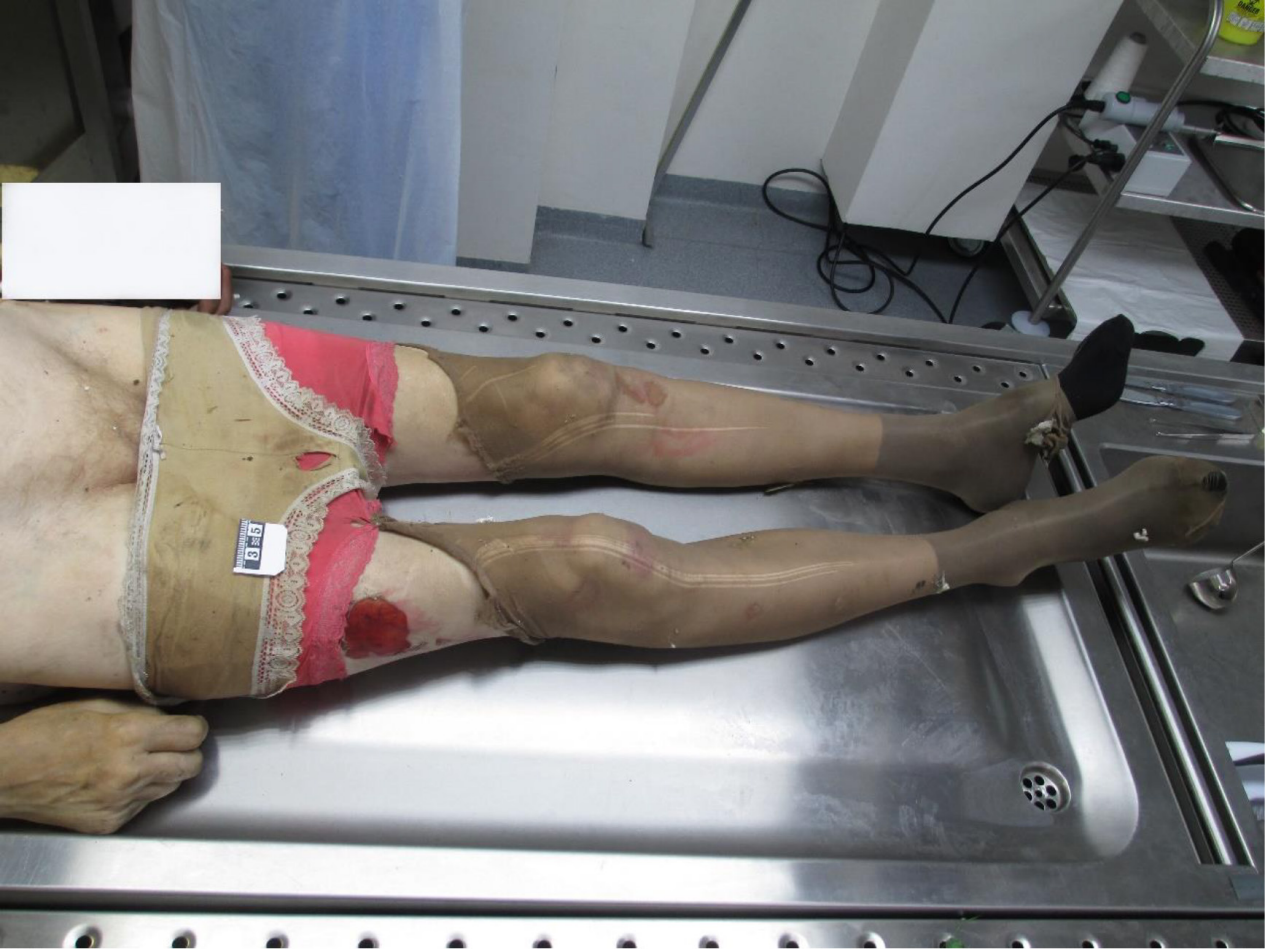
Corpse clothing

**Fig. 2:**
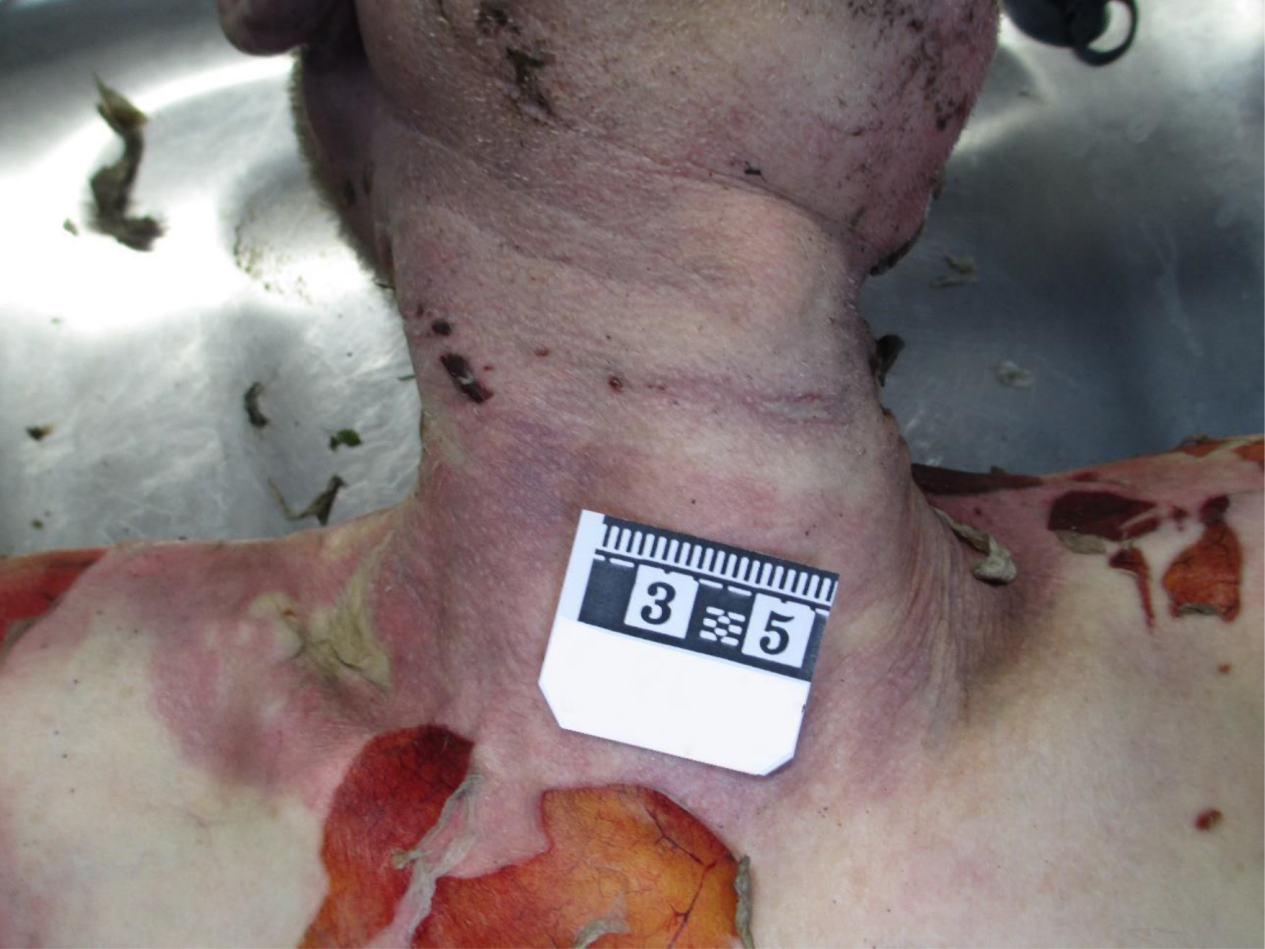
Ligature mark on the neck

A male body was found after the house fire was extinguished. At the scene, the body was found hanging in a noose in the bedroom of the house. Upon external examination, the 65-year-old body was clothed in white sleeveless t-shirts, white and pink women’s underwear, brown women’s pants, and black socks (Fig. 1). The deceased, identified as a European male with a regular build, stood at 182 cm in height. Notably, a significant finding was an open ligature mark on the neck, positioned at a slight slant and extending 1.5 cm below the lower border of the thyroid cartilage (Fig. 2). A detailed examination of the neck tissues was performed, and bruises were found in the sternocleidomastoid muscles of the neck and in the subcutaneous tissue in the projection of the strangulation cord. Carotid arteries on both sides of the neck were examined and tears in their internal membranes were found. The hyoid bone and thyroid cartilage were also evaluated – the hyoid bone and thyroid cartilage were not broken. There were signs of acute pulmonary distension. Blood carbon monoxide was tested – no signs of carbon monoxide poisoning were found. No soot was detected when the airways were opened. The body exhibited a postmortem burn covering 30% of the body surface area, likely due to exposure to high temperatures or flames after death.

The autopsy findings of the body revealed an absence of any other notable injuries. There were no hemorrhages in separated tissues, a peritoneum was intact, and the cranial examination highlighted intact cranial bones and well-differentiated cerebral tissues without hemorrhages. Atherosclerotic changes in the cardiovascular system, edema and hyperemia of internal organs, and steatosis of the liver have been identified. Furthermore, the histological findings exhibited fibrosis of the myocardium, edema, hyperemia of the brain and lungs, hyperemia of kidneys, steatosis of the liver, and hyperemia of the pancreas and adrenal glands. The circumstances of the discovery, data on the scene, autopsy data, and data from additional tests indicate the pathogenesis of death.

Additionally, the toxicology analysis revealed high concentrations of ethanol in blood (3.26 ‰) and urine (4.16 ‰), indicating significant alcohol intoxication.

The individual’s cause of death was established as suffocation due to neck compression by a ligature, supported by the presence of a distinct strangulation-type ligature mark and hyperemia of internal organs, lung, and cerebral edema.

### Case 2

The naked 55-year-old male body of European descent and approximately 184 cm tall, exhibited strong rigor mortis and livor mortis on the posterior regions. There were no visible signs of decomposition or external injuries.

The autopsy findings exposed no internal bleeding. A foreign body was introduced into the anus, at autopsy it was found that the foreign body was in the rectum. Notably, a 2.5 cm rupture in the ileum was detected, caused by a 28 cm plastic tube penetrating the abdominal cavity and intestinal wall. The intestinal loops were covered with fibrin plaque. The reddened, matte, covered with a fibrin plaque, and slightly thickened peritoneum was found. In the peritoneal cavity, there was 300 ml of cloudy, yellowish, purulent, foul-smelling fluid. Based on the degree of development of peritonitis, the minimum time from intestinal perforation by a foreign body could be 3–6 hours. Additionally, atherosclerotic changes in the heart, hyperemia, and edema of internal organs were found.

**Fig. 3:**
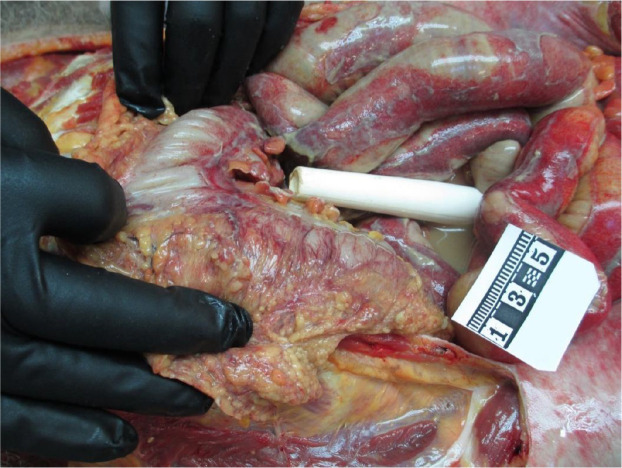
Foreign body in abdominal cavity

The forensic medical diagnosis attributed the main cause of death to the perforation of the ileum by a foreign body – a plastic tube found in the abdomen – resulting in complications including purulent diffuse peritonitis, intoxication, and acute cardiac and respiratory failure, leading to death within 3–6 hours after insertion (Fig. 3). Concurrently, the individual had coronary artery and aortic atherosclerosis, hepatic steatosis, and a renal cyst. Additionally, toxicology findings revealed the absence of ethyl alcohol in the blood but a presence of 0.17‰ in the urine.

The findings of both cases are summarized in Table 1.

**Table 1:** 2 cases, where the death was caused by autoerotic events.

	Case 1	Case 2
*Gender*	Male	Male
*Age*	65	55
*Place*	Home	Home
*Cause of death*	Suffocation	Perforation of the ileum
*Alcohol in blood (‰*)	3.26	Not detected
*Alcohol in urine (‰*)	4.16	0.17

## Discussion

Engaging in sexual activities resulting in death is a relatively rare phenomenon. Many such causes of death are concealed due to family wishes. However, the literature contains numerous documented cases and studies on this topic. These deaths are commonly categorized into autoerotic and internal causes [1, 2]. In both groups, males predominate, with an average age of around 40 for autoerotic deaths and 60 for internal causes [2]. Autoerotic death refers to an accidental incident where an individual dies while using certain tools to enhance sexual sensations. Autoerotic deaths are further classified into typical and atypical cases [3]. Typical cases involve asphyxia and constitute the highest number of deaths [3]. Asphyxia can result from covering the face with objects such as plastic bags, rubber masks, or adhesive tape, hanging, suffocation, chemical substance inhalation (chloroform, cocaine, alkyl nitrites, MDMA, alcohol, toluene, α-PVP, buprenorphine, mephedrone), or respiratory tract obstruction by fluids [2-5, 7-15]. Atypical autoerotic deaths encompass other scenarios, including electrocution, hyperthermia from wearing extra clothing, and traumatic deaths due to foreign bodies inserted in body orifices, which comprise a smaller portion of autoerotic deaths [3, 5, 7, 12, 13, 15]. Often, multiple methods can be combined [4, 5, 7-9, 11, 12, 15].

The most common internal causes of death are cardiovascular diseases [1, 5, 16, 17]. The potential diseases are diverse, including chronic hypertensive cardiovascular disease, aortic coarctation, atrioventricular node tumor, AV node artery fibromuscular dysplasia, air embolism, subarachnoid hemorrhage, right ventricular arrhythmogenic cardiomyopathy, sudden death due to arrhythmia syndrome, aortic dissection, hypertrophic cardiomyopathy, ischemic heart disease, idiopathic fibrosis, idiopathic left ventricular hypertrophy, mitral valve prolapse, myocardial infarction, various channelopathies, and cardiac tamponade [1, 2, 5, 16, 17].

While males predominantly experience sexual activity-related deaths, literature also describes rare cases of female deaths – death during sexual intercourse due to intraplacental choriocarcinoma rupture [6], Bartholin’s gland cyst rupture during sexual intercourse [18], vaginal laceration during sexual intercourse [19], and a woman’s strangulation due to resisting sexual intercourse with a man [20].

## Conclusions

In this case study we presented two case reports on deaths during sexual activities in Lithuania. Deaths during sexual activities are described worldwide, with men being the dominant gender affected by such fatalities. The main cause of autoerotic deaths both globally and in Lithuania is asphyxiation, with foreign body insertion into the body also being a frequent factor. The average age for autoerotic deaths in Lithuania is higher than the global average, ranging from 55 to 65 years. Often, deaths of this nature are concealed due to prevailing societal taboos, like cases where deaths are hidden due to factors like fire accidents or when the deceased did not seek help in time.
